# Continuous Cultivation as a Tool Toward the Rational Bioprocess Development With *Pichia Pastoris* Cell Factory

**DOI:** 10.3389/fbioe.2020.00632

**Published:** 2020-06-25

**Authors:** Miguel Angel Nieto-Taype, Xavier Garcia-Ortega, Joan Albiol, José Luis Montesinos-Seguí, Francisco Valero

**Affiliations:** Department of Chemical, Biological and Environmental Engineering, School of Engineering, Universitat Autònoma de Barcelona, Bellaterra, Spain

**Keywords:** *Pichia pastoris*, continuous cultivation, steady-state omics, physiological characterization, rational bioprocess development, bioreaction kinetics, heterologous protein production, systems microbiology

## Abstract

The methylotrophic yeast *Pichia pastoris* (*Komagataella phaffii*) is currently considered one of the most promising hosts for recombinant protein production (RPP) and metabolites due to the availability of several tools to efficiently regulate the recombinant expression, its ability to perform eukaryotic post-translational modifications and to secrete the product in the extracellular media. The challenge of improving the bioprocess efficiency can be faced from two main approaches: the strain engineering, which includes enhancements in the recombinant expression regulation as well as overcoming potential cell capacity bottlenecks; and the bioprocess engineering, focused on the development of rational-based efficient operational strategies. Understanding the effect of strain and operational improvements in bioprocess efficiency requires to attain a robust knowledge about the metabolic and physiological changes triggered into the cells. For this purpose, a number of studies have revealed chemostat cultures to provide a robust tool for accurate, reliable, and reproducible bioprocess characterization. It should involve the determination of key specific rates, productivities, and yields for different C and N sources, as well as optimizing media formulation and operating conditions. Furthermore, studies along the different levels of systems biology are usually performed also in chemostat cultures. Transcriptomic, proteomic and metabolic flux analysis, using different techniques like differential target gene expression, protein description and ^13^C-based metabolic flux analysis, are widely described as valued examples in the literature. In this scenario, the main advantage of a continuous operation relies on the quality of the homogeneous samples obtained under steady-state conditions, where both the metabolic and physiological status of the cells remain unaltered in an all-encompassing picture of the cell environment. This contribution aims to provide the state of the art of the different approaches that allow the design of rational strain and bioprocess engineering improvements in *Pichia pastoris* toward optimizing bioprocesses based on the results obtained in chemostat cultures. Interestingly, continuous cultivation is also currently emerging as an alternative operational mode in industrial biotechnology for implementing continuous process operations.

## Introduction

In recent years, recombinant protein production (RPP) technology has given rise to a multibillion-dollar market offering products for a wide range of industrial activities such as food, detergent, paper, chemical, cosmetic, and pharmaceutical production. For example, more than 400 RPP-based pharmaceutical products are currently available on the market (Sanchez-Garcia et al., 2016; Vieira Gomes et al., 2018). In parallel, and derived from the RPP technology, metabolic engineering has opened the door to metabolic flux modification and new heterologous reactions. This enable the manufacture of a wide range of products of interest including vitamins, amino acids, ethanol, antimicrobials, enzyme inhibitors, and organic acids. The overall economic impact of RPP and metabolite productions was estimated at around 143.5 billion US dollars in 2014, with upward forecasts until 2020 (Porro et al., 2011; Singh et al., 2017).

Various organisms ranging from bacterial hosts to transgenic animals have been proposed as efficient cell factories for bioprocesses with the mentioned applications. For example, for RPP, most of the products including pharmaceuticals and industrial enzymes are obtained with the main considered workhorses, namely: *Escherichia coli, Saccharomyces cerevisiae* or Chinese hamster ovary (CHO) cells (Sørensen, 2010; Maccani et al., 2014). However, the use of non-conventional yeasts, which combine eukaryotic ability for protein processing and major microbial advantages, have lately started to be considered as promising alternatives. In general, the new choices offer many advantages in terms of pathway requirements, desired product profile, and gross physiology over *S. cerevisiae* (Wagner and Alper, 2016). Such is the case with *Schizosaccharomyces pombe, Hansenula polymorpha* (syn. *Ogataea polymorpha*), *Kluyveromyces lactis, Yarrowia lipolytica*, or *Komagataella phaffii (Pichia pastoris)* (Çelik and Çalik, 2012; Baghban et al., 2019). Actually, at present *Pichia pastoris* (recently classified as *Komagataella* spp.) has arisen as an efficient and versatile cell factory for obtaining a wide spectrum of biotechnological products including recombinant proteins and metabolites of diverse origins (Gasser et al., 2013; Peña et al., 2018).

The key features that make *P. pastoris* an outstanding host for the above-mentioned uses include fast growth at high densities in defined media, the availability of advanced tools for genetic modification (e.g., CRISPR/Cas9 system) and the ability to perform post-translational modifications as well as to secrete the products extracellularly (Vogl and Glieder, 2013; Gasser and Mattanovich, 2018; Weninger et al., 2018). Furthermore, the increasing knowledge on *P. pastoris* metabolism gathered over the last decades, together with the high RPP potential of this yeast, have strongly increased the interest in using this microbial cell factory to obtain a variety of compounds. Thus, strengthening *P. pastoris* as one of the most suitable chassis for the biotechnological industry.

Promoters have proved to be key regulators for RPP processes. For *P. pastoris*, the methanol inducible alcohol oxidase promoter (P_*AOX*1_), a strong and tightly regulated promoter, was the first used obtaining efficient production rates of several proteins of interest (Cregg et al., 1993; Paulová et al., 2012; Ponte et al., 2016, 2018; Lee et al., 2017). Although P_*AOX*1_ affords outstanding RPP levels, using methanol has some relevant drawbacks, especially at production scale processes. These are mainly related with the increased costs and risk of storing and using methanol, as well as the increased oxygen demand and high heat production derived from methanol metabolization (Heyland et al., 2010; Prielhofer et al., 2013; Çalik et al., 2015). These shortcomings have given ground to dedicate important efforts to identify and to develop methanol-free alternatives, which can be classified in two categories: discovery of alternative efficient promoters for *P. pastoris* (P_*GAP*_, P_*PGK*_, P_*THI11*_, P_*PYK*_, P_*SDH*_) (Periyasamy et al., 2013; Landes et al., 2016; Juturu and Wu, 2018; Massahi and Çalik, 2018; de Macedo Robert et al., 2019) and engineering or development of synthetic promoters (Ata et al., 2017; Wang et al., 2017; Prielhofer et al., 2018; Vogl et al., 2018b).

Nevertheless, not only is the promoter selection key in the strain engineering development to construct efficient cell factories, other relevant factors such as host strain background, gene engineering and dosage, protein processing helpers or secretion machinery also have a significant impact on the overall performance of the strain (Ahmad et al., 2014). Furthermore, it is crucial to combine the mentioned factors with an efficient bioprocess development from a bioprocess engineering approach (García-Ortega et al., 2019). For the latter, it should be essential to carry out a characterization producer clone used as a cell factory over different culture conditions to understand its performance and therefore, to design the rational based bioprocess strategies that allows to achieve the optimal performance for industrial bioprocesses (Yang and Zhang, 2018).

To obtain a proper characterization, cells should be kept under constant conditions in terms of both physico-chemical operating conditions as well as their metabolic state (Hoskisson and Hobbs, 2005). In this sense, continuous operation, which was first reported in 1920 (Cooney, 1979), is considered the most simple operational mode for this purpose. Continuous cultures are defined as a fermentation systems in which fresh medium is continuously added to the bioreactor and the components of the culture broth—cells and metabolites included—are continuously removed from the vessel (Fernandes et al., 2015); thereby forcing the cells to proliferate at a fixed rate and in a constant environment reaching the stationary state (Gramelsberger, 2018). The two main types of continuous cultures, chemostat, and turbidostat, are analogous and differ mainly in their cell growth control mechanism (Prielhofer et al., 2013; McGeachy et al., 2019). On one hand, chemostat is controlled through the continuous addition of culture medium in which a single nutrient is present at growth limiting conditions (Peebo and Neubauer, 2018). On the other hand, in turbidostat, the goal is to avoid nutrient limitation while growth is controlled by using an optical sensor which maintains a determined turbidity level by adding fresh medium through a feedback control loop. In this case, the resulting specific growth rate (μ) obtained is close to the maximum specific growth rate (μ_*max*_) of the microorganism used (Gresham and Dunham, 2014; Fernandes et al., 2015).

**Figure 1:**
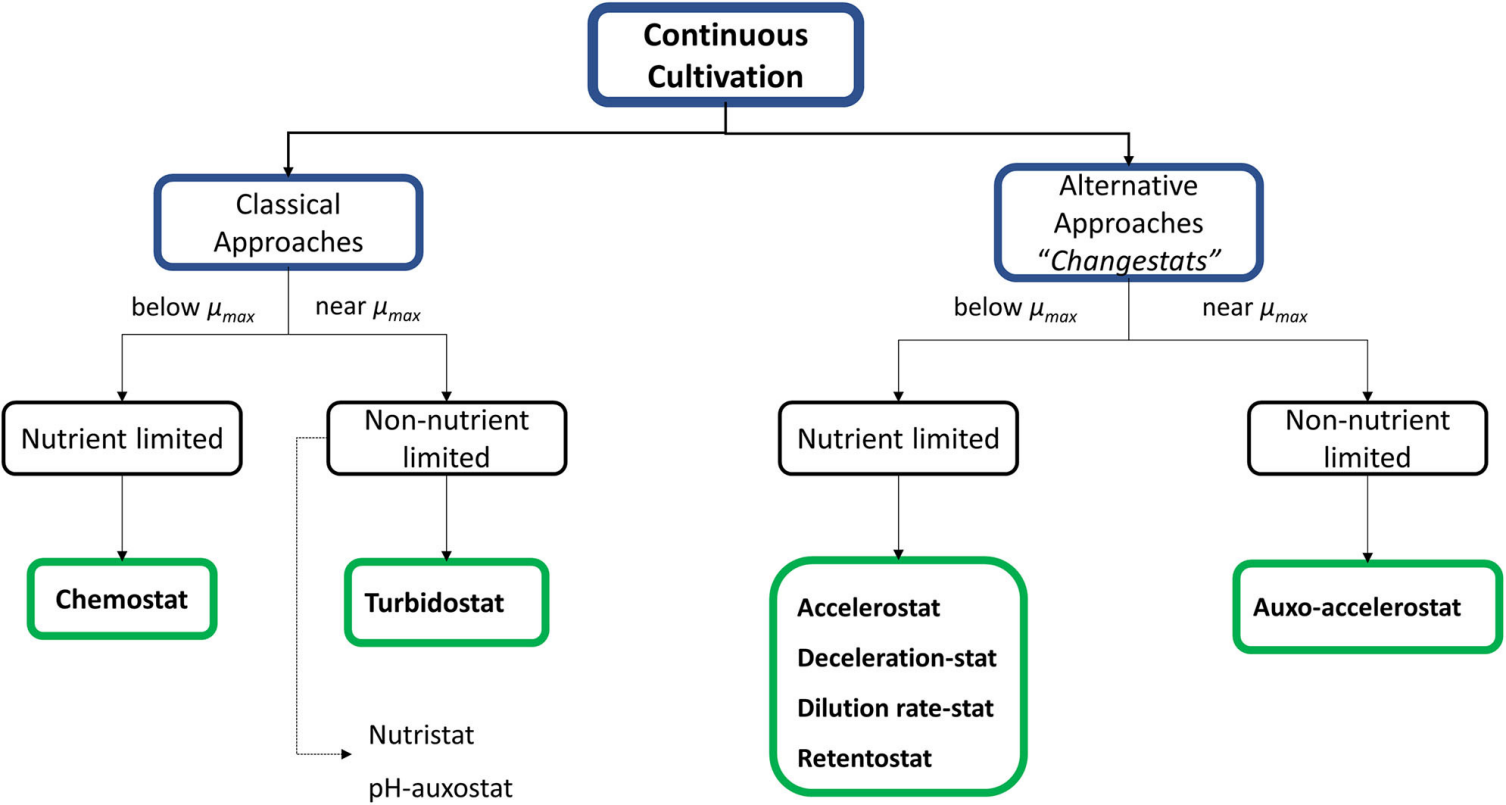
Overview of different continuous cultivation strategies. They are divided in two main groups: the first, corresponding to the “classical” approaches which work at steady state conditions; whereas for the other hand, changestats are variations of classical strategies proposed to obtain similar results but at dynamic conditions allowing faster results.

Additional alternatives such as “changestats” have been proposed as a novel tool where an environmental parameter is continuously changed in a single experiment. These approaches provide quick information about the response of cells in front of determined environmental conditions under what has been called steady state growth space analysis (GSA) (chemostat or turbidostat-based approaches) (Adamberg et al., 2015). Several variants have been described in the literature and have been listed and classified below according to the limitation or not of a nutrient and it is summarized in Figure 1.

Nutrient-limited changestats:

– Accelerostats (A-stats) and deceleration-stats (De-stats), which are used to study the increase or decrease the specific growth rate, respectively (Nahku et al., 2010; Hoekema et al., 2014; Wagenen et al., 2015; Gerritzen et al., 2018).
– Dilution rate stats (D-stats), where an environmental parameter is continuously changed while the dilution rate is kept constant (Kasemets et al., 2003).
– Retentostats, where biomass is retained by means of internal or external filter devices. In prolonged retentostats, the energy derived from the consumed carbon source only allows to sustain the cellular maintenance but can no longer support biomass growth. This allows experiments to be performed at very low or near-zero specific growth rates (Herbert, 1961; Ercan et al., 2015a,b; Rebnegger et al., 2016).

Non-nutrient-limited changestats

– Auxo–accelerostats, allows to examine the effects of smooth environmental changes under nutrient-excess conditions (Nisamedtinov et al., 2008).
– Nutristats, where the dilution rate is a function of the set-point substrate concentration in the bioreactor (Curvers et al., 2002).
– Finally, as special case, adaptastats allow to operate under substrate-limiting conditions but, like turbidostats, work near μ_*max*_ (Tomson et al., 2006).

Although changestats have been presented as promising alternatives to classical continuous cultures, it should be noted that in these systems cells grow in a quasi-steady state because the absence of a total stabilization phase prevents them from reaching steady state (Subramanian et al., 2017). This is the reason why chemostats continue to be the main choice for characterizing microorganisms under substrate-limiting conditions (Ziv et al., 2013). In fact, first reported in 1950 (Monod, 1950), chemostat cultures have proved the best operational choice for precise kinetic and metabolic characterization; also, they have provided the most accurate quantitative understanding at whole-cell level (Hoskisson and Hobbs, 2005). Thus, the empirical knowledge obtained from this characterization allows the determination of several parameters at different conditions, which therefore can be used to identify the optimal culture conditions toward the maximization of product related parameters such as production rates and yields. The relation between cultures and production parameters is detailed through different examples in the following sections. On the other hand, the application of systems biology approaches based on continuous cultures has enabled the development of new highly sensitive analysis tools capable of detecting slight changes at different regulation points, which can be deemed significant when integrating experimental results (Rebnegger et al., 2014).

The following sections describe the basis and uses of continuous cultivation with *Pichia pastoris*. Specifically, it has been focused on carbon-limited chemostat cultures, which according to the literature is the most used strategy for *Pichia pastoris* in continuous operation. The role of chemostat cultures has been reviewed as a powerful tool for the characterization of *P. pastoris* strains with a view to enabling the rational design of engineered cell factories and optimal operational strategies for maximal bioprocess efficiency.

## Continuous Cultivation Provides an Excellent Tool for Systems Biology Research

### Steady-State Omics, a Key Issue in the Renaissance of Research Into Continuous Cultures

The development of high-throughput molecular biology techniques in the post-genomic era has brought a renaissance of continuous cultures. At present, they are used not only in kinetic characterization and adaptive laboratory evolution (ALE) studies (Bull, 2010), but also as a powerful tool for gaining deeper insight into cell behavior thanks to the large amount of robust information they provide. In this sense, continuous cultivations have been used to understand cells response to internal traits such as those observed in engineered clones (using promoters engineering as an example) or to external stimuli such as operational strategies or environmental stress.

**Figure 2:**
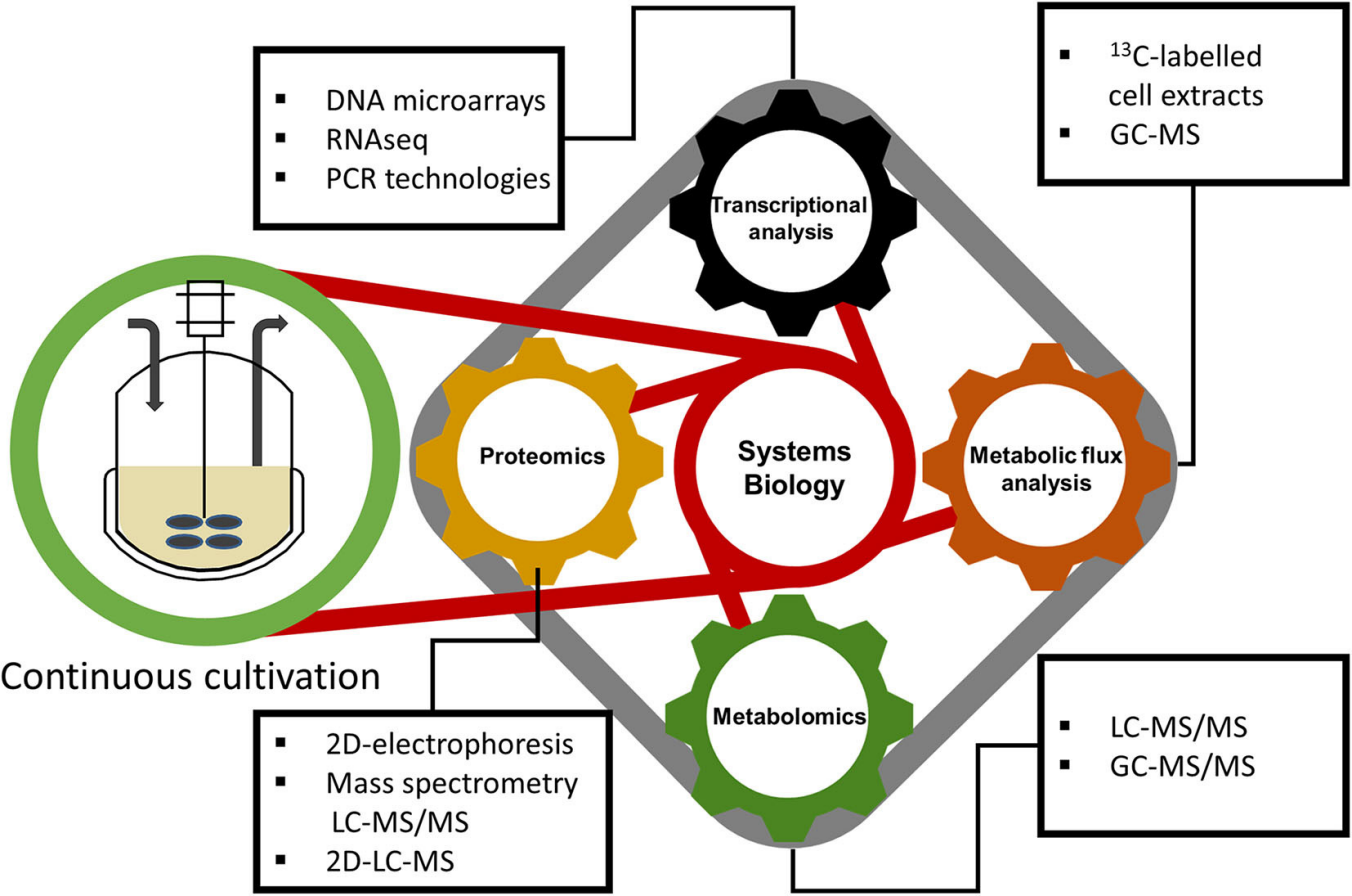
Valuable information that gives continuous cultivation to systems biology for a deep understanding about cell physiology.

Transcriptomic, proteomic, metabolomics, and fluxomic data, can be jointly integrated into a systems biology approach (shown in Figure 2) in order to obtain an all-encompassing picture of a biological system used (Graf et al., 2009). A more detailed description is provided below in the next sections.

### Transcriptional Studies

Transcriptional analysis is usually the first step in systems biology studies as it is also the first stage in the information flow from genomic data (Engstrom and Pfleger, 2017). Gene regulation can be examined by absolute or relative quantification of individual transcripts. Integrating this information enables further interpretation of data and reveals how the complex machinery behind a specific phenotype operates as a result of certain experimental conditions. The insight thus gained can be contrasted with knowledge derived from other structural levels of systems biology.

The contribution of transcriptional regulation to physiological outcomes is strongly dependent on the host employed. Thus, although 27% of all protein-coding genes in human cell lines are transcriptionally regulated (Vogel et al., 2010), the proportion amounts to 50% in *E. coli* and up to 70% in *S. cerevisiae* (Lu et al., 2007). These figures testify to the high significance of interpretations of transcriptional research in yeasts, where *P. pastoris* is taxonomically located.

Transcriptional data are typically acquired in either of two methodologies:

- One involves using specific primers for selected target genes either by real-time quantitative PCR (qPCR) (Landes et al., 2016) or droplet-digital PCR (ddPCR) analyses (Cámara et al., 2019). Both methods provide numerical results and hence quantitative data. Also, they are commonly used to examine the effects on the transcription regulation of certain genes of known sequence.
- However, other high-throughput technology methods such those using microarrays, based qualitative fluorescent detection, and/or RNA sequencing (RNAseq), which quantify the number of transcripts present in a sample by whole transcriptome sequencing, have arisen as helpful workhorses. In fact, these methods provide a robust overview of all transcripts (Baumann et al., 2010; Ata et al., 2018) and are thus especially useful when increased gene coverage is needed.

The selection of the method to be used in each case will be dictated by the specific objectives and information available about the studied system.

Continuous cultivation has proved useful for transcriptional analysis. Specifically, chemostats are frequently deemed the most suitable operational choice for this purpose, as seen with *S. cerevisiae* strains producing either recombinant proteins (Liu et al., 2013) or metabolites (Vos et al., 2015). As shown by several successful attempts described below, chemostat uses could be extended to *P. pastoris*, a similar cell factory related in the taxonomical group of yeasts.

Expression of heterologous proteins and metabolites in *P. pastoris* triggers many changes in cell physiology including stoichiometric drain of energy and cellular resources, or restrictions in the biochemical machinery responsible for bioproduct synthesis (Hesketh et al., 2013; Nocon et al., 2014). Identifying different points of regulation through transcriptional analysis can therefore allow useful information about cell performance to be obtained. Such information could be used to address new targets by strain engineering and to develop innovative, more efficient operational approaches by bioprocess engineering.

Puxbaum et al. (2015) provided a solid discussion about the potential limitations of RPP in *P. pastoris*; they found protein synthesis, folding, and secretion to be the main bottlenecks for the process. In this sense, Hesketh et al. (2013) directly observe stress arising during RPP by expressing under *AOX1* promoter two lysozyme variants differing in degree of misfolding in chemostat cultures. The effect on unfolded protein response (UPR) was examined by using the RNAseq SOLiD platform. In addition, further RT-PCR analysis showed a constitutive splicing of *HAC1* gene transcript across the different phases of RPP induction independent of the lysozyme misfolding level. The importance of this finding is based on the fact that Hac1p corresponds to a key transcription factor in the triggering of UPR response (Graf et al., 2008), and data obtained suggests that UPR is dependent on the transcript regulation of *HAC1* rather than on the splicing process. As a result, the genes *KAR2* and *PDI1*, which are further down the line in UPR, also exhibited high relative expression as revealed by RNAseq transcriptome analysis. Finally, a potential role of untranslated RNA molecules differentially regulated under different expressing conditions was suggested.

On the other hand, endoplasmic reticulum associated protein degradation (ERAD) is one other limiting factor for RPP (Dragosits et al., 2010; Adelantado et al., 2017). UPR and ERAD have been found to jointly control protein folding to a considerable extent and also, generally, heterologous protein synthesis, in *P. pastoris* (Delic et al., 2013; Zahrl et al., 2018). The relevance of both mechanisms as a response to RPP was confirmed by a microarray analysis intended to shed light on the transcriptional upregulation of these mechanisms in *P. pastoris* clones expressing a 2F5 Fab fragment relative to the wild type strain X-33 in chemostat cultures (Gasser et al., 2007).

Furthermore, it has been observed that transcriptional analysis provides useful information for characterizing native and engineered promoters. Thus, Landes et al. (2016) characterized the production of recombinant human serum albumin (HSA) by using an alternative promoter from the thiamine biosynthesis gene *THI11*, which is thiamine-repressible. An initial transcriptional assay on batch cultures using a specific transcriptional analysis with qPCR provided insight into the promoter performance under non-repressive conditions—absence of thiamine—and additional batch experiments allowed to define repression and de-repression effects of the thiamine concentration. Also, chemostat cultures were used to evaluate the performance of the same promoter under carbon-limited conditions at different specific growth rates in order to monitor production rates, and *THI11* and HSA gene transcription. Whereas, the specific production rate (*q*_*HSA*_) improved with increasing specific growth rate (μ), gene transcription had no influence on the latter, indicating a constitutive nature of P_*THI11*_.

In similar work intended to develop alternatives to the methanol-based expression system, Prielhofer et al. (2013) identified six novel promoters through microarray transcriptomics analysis under carbon non-limiting and limiting conditions (batch and chemostat cultivation, respectively). One of the promoters, P_*G*1_, corresponding to a high-affinity glucose transporter (*GTH1*), exhibited strong and tight regulation under carbon-limiting conditions. Also, further characterization of this promoter at variable specific growth rates in chemostat cultures revealed the optimal specific production rate at a dilution rate *D* = 0.07 h^−1^.

As it was observed previously, the useful information derived from the production kinetics of recombinant *P. pastoris* producer clones growing at controlled specific growth rates in chemostat cultures have also enabled further transcriptional characterization and expansion of existing knowledge about the impact of this key parameter for improvement of the cell factory. Thus, Rebnegger et al. (2014) performed a transcriptional analysis using microarrays on *P. pastoris* clone expressing HSA under *GAP* promoter in chemostat cultures to examine the transcriptional response at different μ values. They found *q*_*HSA*_ and μ to be linearly related, and upregulation of ribosomal and translation-related genes in response to higher heterologous protein production. On the other hand, upregulation of UPR and secretory pathways at high μ levels suggested that, although high *D* rates resulted in no limitation in *q*_*HSA*_, they caused heterologous protein production-related stress. Furthermore, marked upregulation of transcriptional regulators involved in stress response as well as a carbon source and nitrogen responsive genes was observed at low μ levels, which suggest an adaptation to the carbon source limitation when the dilution rate was tuned. Finally, consistent with the widely reported Crabtree-negative effect on *P. pastoris* metabolism, mitochondrial transcripts were positively correlated to μ. It is worth mentioning that Crabtree effect corresponds to the respiro-fermentative phenotype widely reported for *S. cerevisiae*, however a similar phenomenon has been observed also in cancer cells (Warburg effect). In order to generate an alternative model for Warburg effect studies, Ata et al. (2018) convert Crabtree negative *P. pastoris* into a Crabtree positive. While determination of Crabtree phenotype was achieved using short- and long-term studies in chemostat cultures, RNASeq transcriptome and ^13^C central metabolism flux analysis were performed in batch cultures. The use of chemostats for further transcriptional and metabolic studies could be proposed to provide new insight of *P. pastoris* as better model than *S. cerevisiae* for Warburg effect studies.

One other interesting use of molecular biology in strain engineering is to integrate multiple target expression cassettes in the *P. pastoris* genome, which usually leads to substantially increased production. Previous reports suggest that gene dosage considerably influences specific productivity (Schwarzhans et al., 2016; Vogl et al., 2018a). Thus, Cámara et al. (2017) evaluated the effect in chemostat cultures growing at μ = 0.10 h^−1^ by using strains with multiple insertions of *Rhizopus oryzae* lipase (ROL) under *AOX1* promoter (0, 1, 2, 4, 8, and 15 copies). As confirmed by *ROL* transcriptional levels in a ddPCR analysis, optimal specific extracellular lipase activity was obtained with the 2C clone. By contrast, transcriptomic analyses with microarrays revealed constraints with increasing *ROL* gene dosage on the transcriptional machinery (specifically, in the methanol expression regulator 1, Mxr1p) (Cámara et al., 2017). This information was used to design new clones carrying several copies of *MXR1* and methanol-induced transcription factor (*MIT1*). As revealed by specific analysis with ddPCR, methanol uptake was found to have a positive effect on the transcriptional response; the effect, however, resulted in no increase in product activity (Cámara et al., 2019).

Not only strain engineering, but also new bioprocess strategies, have led to enhanced RPP. Therefore, better understanding the way cells adapt to different external conditions requires accurate physiological characterization (Baumann et al., 2008; Dragosits et al., 2010; Garcia-Ortega et al., 2016). Based on the positive effect of oxygen-limiting conditions on RPP (Baumann et al., 2008), the impact of these stress conditions was further examined by using transcriptional analysis with microarrays in samples obtained from chemostat cultures under normoxic, oxygen-limited and hypoxic conditions to study the expression of an antibody fragment (Fab) under the constitutive *GAP* promoter (Baumann et al., 2011). Supplementary proteomic and metabolic flux analyses, and lipid-profile analyses (lipidomics) support the findings obtained previously (Baumann et al., 2010; Adelantado et al., 2017). The ensuing information enabled thorough characterization of the oxygen-limiting effects observed in metabolism regulation, which included upregulation of genes associated to the glycolytic pathway, the oxidative branch of the pentose phosphate pathway (PPP), ergosterol and sphingolipid biosynthesis and UPR genes, while the tricarboxylic acid cycle (TCA) revealed transcriptional downregulation.

Osmotic stress has been reported to exert a beneficial effect on RPP. Thus, Dragosits et al. (2010) conducted a transcriptional analysis by using microarrays in combination with chemostat cultures and found increasing concentrations of KCl to result in upregulation of genes involved with UPR, ribosome biogenesis and translation, albeit only in the wild-type clone. Although a Fab-producing clone exhibited higher transcriptional levels than the wild-type strain, such levels were independent of the experimental conditions and no increase in product formation was observed at higher concentrations of KCl. This result suggests that upregulation of these genes as revealed by microarray analysis was previously triggered by a recombinant protein effect and that osmolarity induced no further increase.

Although most transcriptional analyses have been performed in chemostat cultures, some have been conducted in retentostats. Rebnegger et al. (2016) performed retentostat studies to outline the physiology and transcriptional response of *P. pastoris* under this operational condition at near-zero specific growth rates. Among the pool of genes that were differentially expressed in microarray hybridization for transcriptome analysis, upregulation in transcripts involved in alternative carbon source consumption (such as ethanol and methanol), global stress-related transcription factors and with nutrient responses reveal an adaptation of low substrate availability at near-zero growth rates. This finding reinforces the reduction observed in the maintenance coefficient (*m*_*s*_), in comparison to chemostat cultures growing at higher μ. Otherwise, protein synthesis in retentostat cultures exhibited insubstantial transcriptional regulation, so further research, for example with RP-expressing clones, to determine whether the alternative cultures could be useful with *P. pastoris* and to outperform *S. cerevisiae* translational capacity at low μ is proposed.

### Proteomic Studies

According to the central dogma of molecular biology, the protein level falls at the last stage of the information flow (van Hove et al., 2016; Engstrom and Pfleger, 2017). This, however, is no reason to underestimate the importance of the information it provides. Proteomics emerged in the mid-1990s as a new approach to determine the proportion of functional proteins containing information translated into the transcriptome pool. Such information is useful to contrast and supplement data obtained at other levels in systems biology in order to support and strengthen the conclusions of other studies (Szopinska and Morsomme, 2010).

Proteomic analysis with various microorganisms has provided interesting results (Brejning et al., 2005; Rossignol et al., 2009; Arvas et al., 2011; Dragosits et al., 2011). The use of proteomics on *P. pastoris* has been widely used in different cultivation modes including chemostat, batch and fed-batch cultures (Baumann et al., 2010; Dragosits et al., 2010; Pfeffer et al., 2012; Vanz et al., 2012, 2014; Lin et al., 2013), though not so widely as in transcriptional analysis. This section focuses on applications involving continuous cultivation, specifically in chemostats.

The earliest example of proteome characterization was accomplished by Dragosits et al. (2009), who compared the effect of temperature changes on recombinant proteome. The greatest changes were observed at the highest and lowest temperature studied (20 and 30°C, respectively). In addition to increased specific productivity at 20°C, 2D-Gel electrophoresis followed by LC-ESI-QTOF tandem MS allowed the identification of 49 out of 150 spots that exhibited significant differences. Proteomic data revealed that some cellular processes were affected by low temperatures. On the one hand, energy metabolism, oxidative stress response, and protein folding protein levels occurred to a lesser extent; whereas, in amino acid metabolism and RNA/ribosomal biogenesis, an increase of protein levels might account for the positive effect on RPP at 20°C.

Dragosits et al. (2010) conducted a similar study to determine the effect of osmolarity on recombinant *P. pastoris* physiology. Thirty-seven out of the total spots detected, 300 for wild-type and 150 for recombinant clone, were successfully identified with significant changes. These results strengthen the findings of transcriptional analysis as regards energy metabolism and protein folding in both clones. On the other hand, as it was observed in transcriptional analysis, a positive effect on UPR response protein levels was observed at high osmolarity conditions, however it was only in control wild-type strain, whereas it was not the case for recombinant strain where no impact was exerted at different KCl concentrations. The author suggests that although osmolarity not exert a direct effect in producer clone, it could be used for preconditioning cells for RPP, and as outcome it can be considered on improved bioprocess and for generate novel routes for strain engineering.

Rußmayer et al. (2015) interpreted proteomic data at other levels of systems biology and demonstrated symbiosis by using multi-level analysis to expose the cellular processes involved in methanol metabolism. They found good positive correlation between transcriptomic and proteomic data, consistent with the strong transcriptional control over protein abundance in *S. cerevisiae* (Lu et al., 2007). Chemostat cultures on methanol–glycerol mixed feed revealed a positive correlation between protein production and transcripts from methanol metabolism, and also with peroxisome biogenesis. There was, however, no clear-cut relationship between the increased protein levels associated to the translation machinery and cytoskeleton organization, which were not reproduced at the transcriptional level.

The usefulness of proteomic analysis as a supplement to transcriptional and metabolic flux analysis can also be illustrated with the results under hypoxia conditions. Thus, Baumann et al. (2010) conducted an integrative multilevel study and found a strong positive correlation between transcriptional levels and protein data. This result provides support for the assumption that transcriptional regulation is stronger than post-transcriptional regulation in *P. pastoris*. In any case, proteomic data should never be considered irrelevant, particularly in those cases were transcript levels do not match protein expression and understanding the behavior of the cell factory is rather difficult as a result.

### Metabolic Fluxes as Key Physiological Indicators

The physiological status of cells, which is directly reflected in their metabolic phenotypes, is a result of the interplay of various key cell processes and factors such as gene expression, protein production and kinetics, regulation and thermodynamic driving forces (metabolite concentrations). A metabolic phenotype is defined by the actual activities of the metabolic reactions in cells (viz., by reaction rates). Because they are rarely constant, reaction rates are usually determined under steady-state or pseudo-steady state conditions and commonly referred to as “metabolic fluxes,” which are thus key indicators of the physiological status of cells.

Determining metabolic fluxes remains difficult despite major technological advances in quantifying all components involved. In fact, it is not always possible to determine the concentrations of all relevant components; also, *in vivo* values for kinetic parameters are usually unavailable. A number of techniques have thus been developed to quantify metabolic fluxes collectively that are known as “metabolic flux analysis” (MFA) or, simply, “fluxomics.” Many of them have been applied to *P. pastoris* and the most salient are discussed below. Using those techniques for metabolic engineering improves existing knowledge of the physiological properties of biological systems, thus enabling the rational design of cell factories and efficient metabolic engineering cycles.

### Metabolic Flux Analysis

As stated above, MFA is widely used for comprehensive analysis of the physiological effects of environmental changes or genetic engineering modifications. Metabolic fluxes (i.e., metabolic reaction rates in the steady state) provide an excellent operational picture of cell operation. A continuous culture provides a simple, convenient tool for maintaining a stable (in steady state) cell metabolism over long periods of time. Comparing metabolic flux distributions under different conditions allows a deeper understanding of the effects resulting from the genetic or environmental changes.

Although determining metabolic fluxes in continuous cultures has the advantage that cell inputs and outputs are stable, it is still a difficult task due to the large number of reactions taking place simultaneously in each cell. Because in steady state reaction rates are constant, it only requires considering the stoichiometry of the reactions to calculate metabolic fluxes. The mass balances for each metabolite involved in such reactions allow one to construct a linear metabolic model. Those metabolic models with a realistic degree of metabolic detail are known as Genome Scale Metabolic Models (GEMs). Using GEMs in combination with steady-state data should in theory allow one to calculate metabolic fluxes. Unfortunately, the intricate interconnections among metabolites, and the small number of flux measurements typically available (usually cell inputs and outputs only), prevent the accurate determination of most of the metabolic fluxes, which requires the combined use of mathematical models, optimization methods and/or experimental techniques such as ^13^C labeling (Ferrer and Albiol, 2014a,b).

Genome-scale metabolic models have become essential tools for MFA and rational-based strain engineering. These models are mathematically structured knowledge bases containing descriptions of all biochemical reactions, metabolites, and metabolic genes for a specific organism—a Biochemical, Genetic and Genomic (BiGG) knowledge base (King et al., 2016). GEMs can be used in a number of ways such as using their elementary modes or extreme pathways to predict phenotypic function, to improve understanding of the metabolism underlying structure, or using the well-known constraint-based modeling approach (Fong, 2014) to predict function, as examples.

Constructing so detailed BiGG knowledge bases is a continuously evolving process due to the still limited knowledge available on some cell details. The availability of the *P. pastoris* genome sequence allowed the development of the first metabolic reconstructions. Thus, the DSMZ70382 genome sequence, and the sequence from the GS115 strain, allowed the PpaMBEL1254 and iPP668 GEM, respectively, to be produced (Chung et al., 2010; Sohn et al., 2010). Although several improved versions of these *P. pastoris* GEMs have been reported (Ye et al., 2017; Tomàs-Gamisans et al., 2018), this is a continuously evolving field and new or upgraded versions of existing models are expected to appear in the near future.

The continuous cultivation methodology has been used to adjust and validate GEMs. Also, GEMs adapted to specific continuous culture conditions have been used with the latest algorithms and optimization techniques to determine metabolic fluxes under constrains derived from experimental data. This approach is highly useful for interpreting such data and also for designing effective metabolic engineering strategies. Its greatest advantage is that it can be used in any type of continuous culture experiment without the need for a special experimental technique such as ^13^C labeling.

The most common alternative to using an optimization technique for metabolic flux calculations involves obtaining supplementary experimental information. Adding a carbon-labeled carbon source to the input medium of a continuous culture is one common method for this purpose. A typical experiment involves establishing a continuous culture and, once it has reached steady state, replace the input culture medium with another one containing a ^13^C-labeled carbon source. The labeled substrate usually consists of a mixture of molecules labeled at different carbon positions in proportions that are selected according to the expected results. The information to be acquired can be either the distribution of the label once steady state is reached (Tomàs-Gamisans et al., 2019) or changes in the label during the transient labeling period (Heyland et al., 2011; Jordà et al., 2012, 2014b; Nocon et al., 2016). In either case, using this information jointly with steady-state input/output fluxes and a metabolic model allows one to calculate metabolic fluxes under specific conditions. Using GEMs in combination with ^13^C labeling requires solving a very large number of equations. It is thus common to use manually or automatically constructed “context-specific” core models (Tomàs-Gamisans et al., 2019) to facilitate calculations and handling of data. These methodologies have been used in combination with chemostat cultures to examine the impact of heterologous protein production and the effects of different environmental conditions on *P. pastoris* metabolism.

Specific growth rate is one of the basic variables influencing the distribution of metabolic fluxes, and the macromolecular and elemental composition of biomass. This specific rate is easy to regulate in continuous cultures. In *P. pastoris*, the influence of the specific growth rate has been studied by applying ^13^C labeling to chemostat cultures fed with various carbon sources (glycerol, methanol) or their mixtures (e.g., glycerol/methanol) (Jordà et al., 2014a; Tomàs-Gamisans et al., 2018). In general, the results were consistent with what is known as the “growth rate hypothesis” which states that increasing the specific growth rate increases the RNA and protein fractions of cells, thereby boosting protein synthesis and energy production at the expense of other fractions such as carbohydrates.

Biomass composition has also been found to depend on the particular carbon source. Thus, cells grown on methanol contain more protein than those grown on glycerol or glucose (Tomàs-Gamisans et al., 2018). This effect has also been seen with mixed glycerol/methanol or glucose/methanol feeds and can be ascribed to the methanol utilization pathway requiring an expanded metabolic machinery (Jordà et al., 2012, 2014a; Rußmayer et al., 2015). On the other hand, the amino acid composition of cells is virtually independent of their specific growth rate and only differs among carbon sources. Based on the foregoing, metabolic fluxes are calculated by using different biomass composition equations whichever the approach adopted (GEMs included). The environmental conditions also affect metabolic flux distribution. For example, altering the chemostat dilution rate or using a different carbon source alters the distribution of metabolic fluxes; as a result, specific consumption and production rates increase with increasing specific growth rate or by-products such as arabitol are formed (Jordà et al., 2014a; Tomàs-Gamisans et al., 2018). Interestingly, the amount of methanol directly dissimilated into CO_2_ is influenced not only by the specific growth (dilution) rate, but also by the methanol fraction in mixed feeds. Experiments using chemostat cultures have shown environmental factors such as oxygen-limited conditions or low temperatures to have a strong impact on MFA (Mattanovich et al., 2004; Dragosits et al., 2009; Baumann et al., 2010; Carnicer et al., 2012).

The other most extensively studied factor in *P. pastoris* continuous cultures in addition to the environmental conditions is the impact of heterologous protein production on MFA. For this purpose, the effects of different model proteins on metabolic flux distribution relative to their non-productive counterparts is usually compared under identical cultivation conditions. The type of heterologous protein produced and, especially, the amount produced, have proved considerably influential on metabolic flux distributions. Thus, increased heterologous protein production has been shown to shift metabolism toward increased energy generation, reduction equivalents (ATP, NADH, NADPH) and building block production (enhanced pentose phosphate pathway, PPP, or TCA cycles) while reducing biomass yield (Heyland et al., 2011; Jordà et al., 2012, 2014b; Nocon et al., 2016). Increased heterologous protein production has also been found to boost protein misfolding and, ultimately, trigger an unfolded protein response (UPR), thereby further draining resources and shifting metabolic fluxes (Graf et al., 2008). The effects of environmental factors and heterologous protein production on metabolic fluxes have fostered the development of *in silico* strategies to optimize metabolic engineering.

## Bioprocess Characterization With Chemostat Cultures

### Microbial Physiology Studies/Physiological Characterization of the Cell Factory

Increasing the efficiency of a bioprocess (i.e., increasing yields and productivities with high-quality proteins) requires a deep physiological understanding of the cell factory. For the topic discussed in this review, such knowledge is expected to eventually allow the rational development of feasible and competitive bioprocesses with shorter times to market. The overall approach is usually not straightforward due to the complexity of the different interactions occurring at many cell physiology levels. Thus, since the interactions between the cell factory performance and the bioprocess conditions define key parameters of interest (KPI) of the production, an empirical determination of those are required. In this sense, to obtain this kind of information accurately, chemostats are considered the most usual continuous cultivation applied (Hoskisson and Hobbs, 2005). As pointed out in the Introduction section, variations of the continuous operating mode can be alternatively implemented to determine nutrient and environmental effects on metabolism and physiology.

Chemostat cultures provide important advantages over other alternatives such as batch and fed-batch cultures. The advantages arise from the fact that operating under steady-state conditions provides highly robust and reliable data, thus allowing one to assess the effect of a specific parameter while keeping all others constant. Therefore, several cultivation conditions such as medium composition, carbon and nitrogen sources, dilution rates, and others can be investigated to optimize their use. Moreover, some additional strain features and bioreaction kinetics can be thoroughly determined using chemostat cultures. Currently, these parameters are considered of capital importance toward bioprocess optimization for the development of improved industrial processes.

Traditionally, shake-flask cultures have been used to find optimal conditions of physical parameters (pH, T) and concretely, batch or fed-batch cultures to determine oxygen requirements (DO, pO2) However, currently, continuous cultivations should be considered a preferred option to assess the effect of any culture conditions. The fact of working in steady state conditions in whichever cell population is considered homogeneous allows to obtain very accurate and reliable results. In contrast, dynamic processes such as batch of fed-batch cultures are expected to be less robust and present more deviations due to possible drifts in culture conditions. Furthermore, also parameter determination such as kinetic rates and yields is also considered to be more complex in dynamic processes.

### Operating Conditions and Media Optimization

Bioprocess performance is influenced by some physical conditions of cultures including temperature (T), pH and dissolved oxygen (DO). The optimum conditions for production may differ from those for growth due to their effect on several factors including protein-specific characteristics, kinetics and sensitivity of products to proteolytic degradation.

The optimum growth temperature for *P. pastoris* bioprocesses is about 30°C, above 32°C protein expression is suppressed and cell growth rapidly decays (Çalik et al., 2015). However, some authors have suggested that operating at lower temperatures of up to 20°C may significantly improve production of heterologous proteins (Surribas et al., 2007). Decreased operating temperatures have a double but opposite effect. On one hand, the solubility of oxygen is increased and consequently the oxygen transfer rate (OTR) is improved. On the contrary, bioreaction rates such as intracellular reactions, proteolysis and cell growth diminish. Reduced proteolysis at low temperatures is the result of decreased protease activity rather than decreased proteases production (Sirén et al., 2006). Thus, some studies have focused mainly on this cultivation parameter. Data obtained in chemostat cultures on the production of an antibody Fab fragment, indicates that folding stress is generally decreased at lower cultivation temperatures, therefore enabling a higher efficiency for protein processing and secretion (Dragosits et al., 2009). The specific productivity was 3-fold higher at the lower temperature of 20°C, observing also a reduction on the flux through the TCA cycle, jointly with lower oxidative stress response and less presence of molecular chaperones.

Berrios et al. (2017) examined the effect of using co-substrates with methanol in the production of *Rhyzopus oryzae* lipase (Rol) by *P. pastoris* in continuous cultures growing at the same μ but at different temperature (22 or 30°C). Overall, the lower temperature led to lower specific productivities irrespective of the co-substrate used. Strikingly, lowering temperature produced an increase in biomass concentration.

As a conclusion of the studies focused on the T effect, the efficiency of a low cultivation temperature depends on the target protein, so this effect should be studied for each case.

The pH of *P. pastoris* cultures is commonly set at 5.0–6.5 (Looser et al., 2015). pH values above 8 diminish cell viability, and can reduce protein activity and stability (Çalik et al., 2015). However, the optimum pH for RPP depends on the properties of the target protein, especially stability related with the isoelectric point (pI). Since the selection of the optimal pH should also consider the effect on the proteases, a pH value of 5.5 is thus often used to minimize this deleterious effect. In addition, an inappropriate pH can cause some components of the medium to precipitate, which should be avoided by choosing a low value in the working range (Cos et al., 2006).

The dissolved oxygen tension in *P. pastoris* cultivation is typically maintained at 20–30% (air saturation) by having the bioreactor operate at a constant agitation rate and using air or oxygen-enriched air as the inlet gas in order to ensure fully aerobic conditions (especially in high-cell density cultures). Chemostat cultures have been used to examine the boosting effect of oxygen-limiting conditions on RPP. For example, Garcia-Ortega et al. (2017) compared different steady-states under oxygen-limiting conditions and found the optimum setting to provide a 3-fold increase in a specific production rate of the recombinant protein.

Using as simple and inexpensive media as possible is essential to reduce fermentation and protein purification costs for efficient RPP. The most common media for *P. pastoris* cultivation have been reviewed (Cos et al., 2006; Sreekrishna, 2010). However, some of them present important problems such as unbalanced composition, formation of precipitates, high ionic strength, etc.

One of the critical points in formulating cultivation media is the nitrogen source. In some cases, nitrogen is only supplied with the feed medium; in others, it is added when pH control is required. Nitrogen starvation produces an increase in protease activity which can be avoided by increasing the initial/inlet concentration of the nitrogen source. Protease inhibitors provide, in general, a more expensive alternative method to the use of protease-deficient strains in order to decrease the presence of proteolytic activity in culture broth (Shi et al., 2003; Sinha et al., 2005).

Cankorur-Cetinkaya et al. (2018) developed a new interesting medium formulation that provide an appropriate balance between cell growth and product formation in the synthesis of human lysozyme (HuLy) and the anti-idiotypic antibody 3H6 Fab in chemostat cultures of *P. pastoris*. Tyrosine supplementation increased productivity, but tryptophan addition had virtually no effect; also, phenylalanine addition increased HuLy expression but decreased 3H6 Fab expression.

The effect of biotin deficiency on the growth of *P. pastoris* producing a recombinant glycosylated avidin was studied in chemostat culture. Replacing biotin with aspartic acid and oleic acid, although they had a growth promoting effect, wash-out happened and avidin productivity decreased. The addition of small amounts of biotin provided stable chemostat cultures on methanol and enabled production of biotin-free avidin (Jungo et al., 2007b).

The composition of the cultivation medium also has a direct impact on osmolarity. Dragosits et al. (2010) examined the effect of osmolarity on cell physiology in wild-type and Fab fragment-producing strains of *P. pastoris* grown in carbon-limited chemostat cultures. Neither total protein nor specific Fab production was affected by osmolarity. However, proteins involved in energy metabolism and folding processes were affected by an increased osmolarity (especially with the wild-type strain). Also, a high osmolarity decreased biomass yield due to the increased energy maintenance requirements.

### Carbon Sources

The main substrate for a recombinant production bioprocess is usually selected in terms of the promoter regulating the expression. With *P. pastoris* and constitutive promoters such as P_*GAP*_, glycerol and glucose are widely used as carbon sources because they result in high specific growth rates that are usually accompanied by adequate specific production rates (Garcia-Ortega et al., 2013). In P_*AOX*1_-driven systems, however, methanol is often the sole carbon source and inducer of protein expression (Barrigón et al., 2015).

*Pichia* strains exhibits a different phenotype regarding methanol assimilation. For Mut^S^ phenotype, cell growth is considerably slow relative to the standard strains, which are designated Mut^+^ strains. Cell growth and productivity in bioprocesses involving Mut^S^ strains can be improved by using mixed substrates in combination with a co-feeding strategy (Arnau et al., 2010, 2011). This increases the total concentration of the carbon source and the amount of energy supplied reducing process times, as well as reducing heat production and oxygen requirements relative to Mut^+^ bioprocesses. Glycerol and sorbitol are the most commonly used co-substrate jointly to methanol (Jungo et al., 2007a,c), although in repressed classical systems, glycerol may lead to lower specific production rates (Arnau et al., 2011; Potvin et al., 2012). New, unrepressed promoters allow glycerol and glucose to be used in combination with methanol to substantially increase RPP efficiency (García-Ortega et al., 2019).

The continuous strategy provides a powerful tool for assessing the potential impact of using co-substrates. The design of this system is usually based on the selection of a dilution rate (D) far enough from μ_*max*_, which should ensure that no accumulation of substrates apart from methanol is produced. Moreover, the methanol concentration used should be high enough to allow efficient induction of the target protein, but not so high as to inhibit cell growth or protein production.

D'Anjou and Daugulis (2001) used a substrate mixture of methanol and glycerol with a Mut^+^ strain for P_*AOX*1_-driven heterologous production of a sea raven antifreeze protein. The specific product formation rate (*q*_*P*_) and product-based volumetric productivity (Q_V_) increased throughout the range of dilution rates used (0.01–0.10 h^−1^). Similarly, Boze et al. (2001) studied the production of recombinant porcine follicle-stimulating hormone (rFSH) with mixed substrates of sorbitol and methanol. Neither protein production nor Q_V_ or *q*_*P*_ increased relative to the use of methanol as sole carbon source at the same dilution rate (0.01 h^−1^). However, the substrate concentration ratio was not optimized in either study.

Jungo et al. (2007a) studied the influence of the methanol fraction in the feed medium on recombinant avidin productivity and specific alcohol oxidase activity by using a glycerol–methanol mixed substrate to grow a *P. pastoris* Mut^+^ strain expressing recombinant avidin at a constant dilution rate *D* = 0.06 h^−1^. With methanol fractions higher than 0.6 C-mol C-mol^−1^, *q*_*P*_ was like cultures performed with methanol as sole carbon source.

Canales et al. (2015) used the production of *Rhizopus oryzae* lipase (Rol) as a model system to investigate the application of methanol-glycerol feeding mixtures in the *P. pastoris* Mut^+^ producer clone. Cultures were grown in a simple chemostat system and response surface methodology was used to evaluate the effects of D and the methanol/glycerol ratio in the feed as experimental variables. The optimum conditions provided *q*_*P*_ values similar to, and Q_V_ values higher than those of processes using methanol alone as substrate. Furthermore, Berrios et al. (2017) compared the effect of glycerol and sorbitol as co-substrates for methanol in continuous cultures growing at the same rate. Higher Q_V_, but lower *q*_*P*_ and volumetric methanol consumption rates were observed using glycerol as a co-substrate at either 22 or 30°C.

Paulová et al. (2012) used a mixture of glucose and methanol in continuous cultures of a Mut^+^ *P. pastoris* strain to obtain recombinant trypsinogen. No repressive effect of glucose on methanol was observed under conditions far enough from μ_*max*_. The product was synthesized throughout the D range, with enhanced productivity at intermediate D values that was significantly higher than with methanol alone in the feed.

Niu et al. (2013) used a co-feeding strategy based on a methanol–sorbitol mixture with the aim of reducing the high oxygen demand at large-scale operation. Thus, they used transient continuous cultures of a Mut^+^/P_*AOX*1_-lacZ strain at a fixed dilution rate. While a linear change of methanol fraction was conducted, cell growth and metabolism, including O_2_ consumption, CO_2_ and heat production were analyzed. Based on the results, decreasing the methanol fraction in the feeding medium reduced the specific oxygen uptake rate and maintained maximal ß-galactosidase *q*_*P*__._ Production was optimal with methanol fractions over the range of 0.45–0.75 C-mol C-mol^−1^.

Although glucose and sorbitol have also been tested as co-substrates jointly with methanol for Mut^+^ phenotype, glycerol is the most selected co-substrate to improve the productivity and to reduce the specific oxygen uptake rate and heat generation in continuous cultures in comparison with the sole use of methanol as a substrate. However, the methanol:glycerol ratio and the operating dilution rate must be empirically optimized for each target protein.

### Bioreaction Kinetics

The physiological characterization of the producer strains, production rates and yields included, is currently considered critical for bioprocess development. Environmental factors affect cell factories in terms such as rearrangement of central carbon, amino acid metabolism and other basic functions, all of which have a direct impact on cell growth, folding stress, and vesicular transport, and also potential implications in protein secretion and other phenomena influencing production rates and yields (Baumann et al., 2010; Zahrl et al., 2017).

Continuous cultivation is the most frequently used operational mode to obtain accurate physiological data for reliable characterization. By contrast, fed-batch cultures are usually deemed less robust, and more laborious and time-consuming (García-Ortega et al., 2019). Also, stress conditions associated to high-cell density cultures can eventually occur and render them unsuitable for accurate physiological studies.

On the other hand, the use of dynamic process conditions for fast physiological strain characterization in order to reduce experimental time associated to conventional fed-batch and/or continuous cultivation has been reviewed (Spadiut et al., 2013). Moreover, the use of transient continuous cultures in order to reduce the time-consuming steady-state experiments was also implemented in the quantitative study of using mixed feeds (Jungo et al., 2007a).

Table 1 summarizes the continuous cultures used so far to characterize the bioreaction kinetics involved in some RPP processes with *P. pastoris*. The table shows the target protein, strain, promoter, substrate, operating strategy, and product formation kinetics. As can be seen, chemostat cultivation was the norm, but alternative strategies based on S-stats (nutriostats) (Curvers et al., 2002) or DO-stats were also used (Yamawaki et al., 2007).

**Table 1:** Summary of continuous cultures used as a tool toward the rational based bioprocess development with *P. pastoris* cell factory.

**Protein expressed**	**Strain**	**Promoter**	**Substrate**	**Continuous strategy**	**Production kinetics**	**Optimization approach**	**References**
Fab fragment (anti-HIV antibody 2F5)	X-33	*GAP*	Glucose	Chemostat	Growth-coupled μ-saturated	Optimized feeding from continuous data	Maurer et al., 2006
Fab fragment (anti-HIV antibody 3H6)	X-33	*GAP*	Glucose	Chemostat	Growth-coupled μ-saturated	Optimized feeding from continuous data	Buchetics et al., 2011
Fab fragment (anti-HIV antibody 2F5)	X-33	*GAP*	Glucose	Chemostat	Growth-coupled	Optimal D at continuous mode	Garcia-Ortega et al., 2016
hGM-CSF (human)	GS115 Mut^+^	*GAP*	Glucose	Chemostat	Linear	Fed-batch designed from continuous data	Khasa et al., 2007
phytase	X-33	*GAP*	Glucose	Chemostat	Linear	Fed-batch designed from continuous data	Tang et al., 2010
srAFP	GS115 Mut^S^	*AOX*	Glycerol/methanol	Chemostat	Growth-coupled	Fed-batch designed from continuous data	D'Anjou and Daugulis, 2001
°scFv	GS115 Mut^+^	*AOX*	Methanol	DO-stat S-stat	Bell shaped μ-saturated	Trade-off between fed-batch and continuous	Yamawaki et al., 2007
hCTRB	GS115 Mut^+^	*AOX*	Methanol	Nutriostat	Linear	Optimal D at continuous mode	Curvers et al., 2002
rHV2	GS115 Mut^+^	*AOX*	Methanol	Chemostat	Bell shaped	Fed-batch designed from continuous data	Zhou and Zhang, 2002
Porcine trypsinogen	X-33	*AOX*	Glucose/methanol	Chemostat	Bell shaped	Optimal D at continuous mode	Paulová et al., 2012
Avidin	GS115 Mut^+^	*AOX*	Methanol/glycerol/ sorbitol	Chemostat	Linear	Fed-batch designed from continuous data	Jungo et al., 2006, 2007a,c
CalB	X-33	*PGK*	Glycerol	Chemostat	Linear	Trade-off between fed-batch and continuous	de Macedo Robert et al., 2019
Serum albumin (human)	X-33	*THI11*	Glucose	Chemostat	Growth-coupled	Optimized feeding from continuous data	Landes et al., 2016

The most common types of kinetics reported for cell growth (μ), substrate uptake (*q*_*S*_), and product formation (*q*_*P*_) are Monod for cell growth, *Pirt*'s as a maintenance energy model and *Luedeking–Piret* as linear μ-dependence for substrate uptake and product formation, respectively. However, saturation patterns, non-monotonic behaviors or bell-shaped types have also been observed, especially in substrate-inhibited systems for cell growth and non-constitutive RPP (Barrigón et al., 2015).

The kinetics of oxygen uptake, carbon dioxide production, and substrate uptake can usually be fitted to a linear model thanks to the rather linear relationship between specific rates and the specific growth rate. These have been widely discussed in several studies on chemostat cultures of *P. pastoris* (Maurer et al., 2006; Rebnegger et al., 2014; Looser et al., 2015; Garcia-Ortega et al., 2016).

Because production kinetics is influenced by a number of physiological interactions, the overall balance of the different steps from gene transcription to product secretion is usually considered instead. As discussed by some authors, the correlation between *q*_*P*_ and μ is specific for each case, so it must be determined experimentally for each individual strain and protein under fixed operating conditions (Looser et al., 2015). The data of Table 1 confirm that production kinetics is not only influenced by the promoter used with each strain but also, often, protein-specific. For this reason, the most widely used expression systems (viz., that driven by methanol-inducible P_*AOX*1_ and the constitutive glycolytic P_*GAP*_) usually exhibit different production kinetics. Thus, the P_*AOX*1_-driven expression system often exhibits non-monotonic kinetics, possibly as a consequence of the metabolic burden resulting from methanol uptake and/or protein processing, and from secreting limitations due to outstanding expression levels (García-Ortega et al., 2019). In this situation, maximizing bioprocess efficiency entails using intermediate μ values far from μ_*max*_. On the other hand, a growth-coupled increasing correlation between μ and *q*_*P*_ has been observed in constitutive expression systems relying on glycolytic promoters such as *GAP* or *PGK* (Rebnegger et al., 2014; Garcia-Ortega et al., 2016; de Macedo Robert et al., 2019), and also with the thiamine sensitive *THI11* promoter (Landes et al., 2016), *q*_*P*_ peaking at higher μ values closer to μ_*max*_.

For most proteins, however, *q*_*P*_ is related to μ by a growth-coupled production model (D'Anjou and Daugulis, 2001; Rebnegger et al., 2014; Garcia-Ortega et al., 2016) and even, possibly, by a *Luedeking–Piret* or μ-linear model (Curvers et al., 2002; Jungo et al., 2006; Khasa et al., 2007; Tang et al., 2010). Other, partially growth-dependent, exhibit a μ-saturated production pattern (Maurer et al., 2006; Buchetics et al., 2011) or fit a bell-shaped model (Yamawaki et al., 2007). For example, Yamawaki et al. (2007) found *q*_*P*_ for scFv to exhibit either a saturation behavior on μ or bell-shaped kinetics depending on the operational mode used (continuous or fed-batch cultivation). For other proteins, studies performed in chemostat (Paulová et al., 2012) or fed-batch cultures (Zhou and Zhang, 2002; Zhang et al., 2005) have shown them to fit μ non-monotonically increasing or bell-shaped models.

In Table 2 a comparison of *q*_*P*_ and Y_P/X_ according to the μ reached in continuous and fed-batch bioprocesses is presented. This table includes results obtained with different target proteins, promoters, gene dosage, substrates, and feeding strategies. In continuous mode, Y_P/X_ normally decreases when μ increases, but in contrast *q*_*P*_ increases along with μ, except in the production of hSA where Y_P/X_ increases when μ increases, which is probably associated with the high gene dosage (6 copies) (Landes et al., 2016). In general, this linear behavior between *q*_*P*_ and μ is related with the commonly observed μ-dependence in *P. pastoris* bioprocesses, which indicates that the product formation is growth-dependent.

**Table 2:** Comparison of specific production rates (*q*_*P*_), product yields (Y_P/X_), and volumetric productivities (Q_v_) between continuous and fed-batch operating modes with different proteins produced, promoters/gene dosage, substrates, specific growth rates (μ), and feeding strategies used.

				**Continuous**				**Fed-batch**				**References**
**Protein**	**Promoter/gene dosage**	**Substrate**		**μh^**−1**^**	***q_***P***_*AU/(g**_X_·**h)**	**Y_**P/X**_AU/g_**X**_**	**Q_v_AU/(L·h)**	**μh^**−1**^**	***q_***P***_*AU/(g**_X_·**h)**	**Y_**P/X**_AU/g_**X**_**	**Q_v_AU/(L·h)**	
Crl1	P*_*GAP*_* Sc	Glucose		0.056 0.081 0.136	78.2 89.1 126	1,400 1,093 930	1,924 2,410 3,108	0.048 0.087 0.133	77 149 292	1,610 1,700 2,190	2,195 3,246 10,442	Nieto-Taype et al., 2020
Crl1	P*_*AOX*_* sc	MetOH		0.020 0.050 0.079	118 271 310	6,017 5,398 3,939	2,291 5,564 6,874	0.028 0.053 0.084	364 588 326	13,155 11,299 3,911	2,291 5,564 6,874	Garrigós-Martínez et al., 2019
CalB	P*_*PGK*_* Sc	Glucose		0.050 0.090 0.160	20.4 38.4 59.1	449 423 377	449 864 1,331	0.060 0.110 0.140	19.4 47.5 52.3	323 423 377	402 796 868	de Macedo Robert et al., 2019
Phytase	P*_*GAP*_*	Glucose		0.050 0.150 0.300	480 820 1,420	9,600 5,500 4,700	nd nd nd	DFP 0.118 DFP 0.125 DFP 0.125	567 586 628	4,800 4,680 5,020	20,900 17,400 18,700	Tang et al., 2010
**Protein**	**Promoter/gene dosage**	**Substrate**		***μ*****h**^**−1**^	***q**_***P***_***μg/(g_X_·h)**	**Y**_**P/X**_**mg/g**_**X**_	**Q**_v_**mg/(L·h)**	***μ*****h**^**−1**^	***q**_***P***_***μg/(g_X_·h)**	**Y**_**P/X**_**mg/g**_**X**_	**Q**_v_**mg/(L·h)**	**References**
Human FAB 2F5	P*_*GAP*_* sc	Glucose		0.050 0.100 0.150	17.2 32.4 45.7	0.33 0.32 0.30	0.34 0.70 1.04	0.050 0.101 0.146	7.7 25.7 35.2	0.15 0.25 0.24	0.24 0.64 0.73	Garcia-Ortega et al., 2013, 2016
Human FAB 2F5	P*_*GAP*_* sc	Glucose		0.060 0.100 0.150	25.1 34.0 41.4	0.42 0.34 0.28	0.62 0.94 1.14	CFR OFR	10.0 30.0	0.48 0.48	0.39 0.67	Maurer et al., 2006
Human FAB 3H6 CLB2	P*_*GAP*_* sc	Glucose		0.190 0.050 0.100 0.150	45.6 48.1 60.5 66.1	0.24 0.96 0.60 0.44	1.38 1.32 1.66 1.82	OFR	10	0.45	nd	Buchetics et al., 2011
rHV2	P*_*AOX*_*	MetOH 0.4 g/L		0.017	nd	nd	nd	0.01	0.03	3.0	nd	
		MetOH 0.6 g/L		0.020	nd	nd	nd	0.02	0.20	9.8	nd	Zhou and Zhang, 2002
		MetOH 1.5 g/L		0.040	nd	nd	nd	0.04	0.08	2.0	nd	
							CLC 0.018	0.17	9.6	14		
hSA	P_**THI*11*_ 6 copies	Glucose		0.050 0.075 0.100 0.125 0.150	0.003 0.017 0.050 0.119 0.180	0.06 0.22 0.50 0.95 1.20	0.05 0.30 1.00 2.38 3.60	0.150	0.023	0.88	2.60	Landes et al., 2016
Avidin	P*_*AOX*_*	^*^MS-MS (% Methanol C-mol)	6 25 43 65 100	0.03 0.03 0.03 0.03 0.03	0.003 0.013 0.016 0.016 0.016	0.11 0.42 0.54 0.52 0.54	0.03 0.13 0.16 0.13 0.12	0.027	0.049	1.78	3.24	Jungo et al., 2007c

*SC: Single Copy; nd: not determined; All the fed-batch cultures were carried out by implementing open-loop feeding strategies except: DFP, Different Feeding Profile; CFR, Constant Feed Rate; OFR, Optimized Feed Rate; CLC, Closed Loop Control*;

* *Mixed-substrates Methanol-Sorbitol. Some values have been estimated from the values indicated in the references or directly from the figures. For rHV2, hSA, and avidin, q_P_ is expressed as mg/(g_x_·h)*.

When the data obtained in both, continuous and fed-batch mode are compared, the analysis is often difficult because not all the examples have been studied for coincident μ'*s*. However, the *q*_*P*_ behavior is quite similar for the two operating modes, only for the case of rHV2 and Crl1 production regulated by P_*AOX*1_, which *q*_*P*_ presents a maximum at intermediate μ for the fed-batch cultivation not following a product formation growth-dependent pattern. On the other hand, there is not a common trend in reference to Y_P/X_ behavior in front of μ, observing different positive and negative linear relationships, bell-shaped and saturation patterns.

In terms of absolute values, only in the case of Fab 3H6 and hSA production, the specific production rate (*q*_*P*_) and the Y_P/X_ values were always higher in continuous mode at similar μ comparing to those obtained in fed-batch cultivation.

Additionally, since productivities could be one of the main performance criteria from an industrial point of view, volumetric productivity (Q_V_) has been also included in Table 2. Nevertheless, the comparison could not be made in a suitable way because the biomass concentration and the reactor volume may not be equivalent in the two operating modes. Moreover, for a proper comparison it had to be also considered a time window of several weeks in order to take into account the unproductive time between consecutive fed-batches. For instance, taking these factors into consideration, overall CalB production was reported as 5.8 times greater in continuous culture (de Macedo Robert et al., 2019).

The results of this experimental approach, which is mostly performed in chemostat cultures, provide a wealth of knowledge to tailor rational strategies with a view to optimizing bioprocess operating conditions for increased production rates and yields. Although production patterns are expected to be similar when using the same expression system to produce different target proteins, the outcome usually depends on both. Therefore, optimal bioprocess development requires using similar experiments not only to maximize production rates, but also to identify the most suitable conditions for testing other strains with industrial potential. Chemostats therefore provide an effective platform for easily characterizing production strains and elucidating production kinetics by the obtention of accurate and robust data.

### Bioprocess Optimization and Process Development

Most of the bioprocesses currently used for recombinant protein or metabolite production are performed in the fed-batch mode on the grounds of the high cell concentrations it affords, and the increased amounts of product obtained as a result. Therefore, using the data obtained from chemostats with fed-batch cultures requires that the latter operate in a pseudo-stationary state or balanced growth in order to maintain the overall μ value rather constant during the process. This can be accomplished by using a fed-batch strategy based on pre-programed exponential feeding derived from mass balances. Under these conditions, if μ is kept constant and the biomass-to-substrate yield rather constant throughout the cultivation period, the substrate can be assumed to reach a pseudo-stationary state.

Table 1 summarizes works with continuous cultures as a tool for further rational process development based on the physiological characterization of the producer strains. As can be seen, three main approaches have been used for optimal RPP. One involves identifying the optimum *D* conditions under continuous operation for key parameters of interest (KPIs) including productivities (Curvers et al., 2002; Paulová et al., 2012). Another uses a trade-off between fed-batch and continuous operation for yields or productivities depending on the particular design or performance criteria (Yamawaki et al., 2007). The third is a sequential approach to bioprocess design (i.e., fed-batch operation is designed and implemented on the basis of production kinetics or productivity data obtained under continuous operation) (Zhou and Zhang, 2002; Jungo et al., 2007a,c; Khasa et al., 2007; Tang et al., 2010).

Garcia-Ortega et al. (2016) characterized the recombinant production of Fab fragment 2F5 driven by the constitutive promoter P_*GAP*_ in chemostat cultures. A positive effect of high μ levels on productivity was observed, which confirmed that production was growth-coupled.

de Macedo Robert et al. (2019) compared *Candida antarctica* lipase (CalB) production with the constitutive promoter *PGK* in continuous and fed-batch cultures. They found volumetric and specific productivity to peak at the highest μ levels, which indicated a direct correlation between growth and production.

Production kinetic profiles for other proteins have also been obtained in other continuous cultures that were used as references to develop optimal feeding strategies adjusted to cell factory performance. The result was a marked increase in production in terms of volumetric productivity and product titer for some Fab fragments (Maurer et al., 2006; Buchetics et al., 2011) and human serum albumin (Landes et al., 2016).

D'Anjou and Daugulis (2001) used glycerol–methanol mixtures as co-substrates for continuous cultures to obtain sea raven antifreeze protein (srAFP). They elucidated the relationship of μ to the yield on methanol, *q*_*S*_ on methanol and *q*_*P*_ and then used data collected under continuous operation to predict cell growth and RPP, as well as to develop an exponential feeding strategy for fed-batch cultures with the same two carbon sources. The outcome was increased product yield and productivity relative to a heuristic approach.

## Is Continuous Cultivation a Real Alternative for its Use in Industrial *Pichia Pastoris* Processes?

### The Transition of Industrial Biotechnology From Batch/Fed-Batch Mode to Continuous Manufacturing

The main current trend in bioprocess optimization is to move away from standard, fixed protocols toward concepts enabling the operator to adapt particular recommendations to any specific clones, strains or bioreactors (Looser et al., 2015). This requires combining effective strains and bioprocess engineering approaches for optimal results, and also avoiding complex media to reduce costs and facilitate downstream processes with a view to increasing economic viability of the bioprocess (Sreekrishna, 2010; Potvin et al., 2012). In this sense, due to the relevant advantages of the continuous manufacturing, as once pioneered in the oil and chemical industry, many industrial bioprocesses are expected to eventually evolve from batch or fed-batch to continuous processes (Croughan et al., 2015).

Currently, continuous biomanufacturing processes based on the application of continuous fermentation are being carried out in different industries including ethanol, lactic acid, and numerous biopharmaceutical productions. Mears et al. (2017) discussed some of these examples performed with continuous microbial systems at industrial scale to evaluate the expected wide implementation in biopharmaceutical biomanufacturing processes. Actually, the US FDA has encouraged the development of continuous processing for biopharmaceuticals manufacturing (Croughan et al., 2015). In this sense, it is worth mentioning that Novo Nordisk implemented the continuous insulin production with the yeast *S. cerevisiae* since the 1990's (Peebo and Neubauer, 2018). And currently, several commercial biopharmaceuticals such as monoclonal antibodies or therapeutic proteins are recombinantly produced in continuous perfusion cultures of mammalian cell lines (Mears et al., 2017).

Industrially, switching from batch to continuous operation can have several advantages such as reduced processing costs, increased productivity, and product quality, and the ability to integrate upstream and downstream in a continuous manner (Cankorur-Cetinkaya et al., 2018). However, bottlenecks in industrial bioprocesses are generally product-specific and must be identified on a case-by-case basis with provision for economic constraints (Yang and Zhang, 2018).

Continuous cultures growing under steady-state conditions are, in theory, more effective in this mode than are batch or fed-batch cultures. In this mode, which is usually performed at a constant μ, most of the factors involved in the RPP as well as a large fraction of the transcriptome, proteome and fluxome can be considered constant, since it has been reported that they are coordinated by μ (Peebo and Neubauer, 2018). Therefore, continuous cultivations where μ can be easily fixed, simplifies significantly the interpretation and control of the bioprocess. In this way, bioprocesses should be easier to characterize, understand, control and maintain under optimal conditions over long periods. Furthermore, the lag and dead times, intrinsically associated to batch and fed-batch operation, result in considerably diminished productivity. Hence, continuous biomanufacturing processes allow reducing running costs, minimizing equipment size, integrating upstream and downstream steps, increasing product quality, ensuring constant product recovery, and making processes scalable are additional advantages of continuous cultures over batch and fed-batch cultures (Peebo and Neubauer, 2018).

However, continuous cultivation also has some drawbacks arising from the need to ensure long-term stability and sterility in the cultures, and to shorten average long development times (Croughan et al., 2015). Specifically, the genetic instability of recombinant strains may limit the length of RPP bioprocesses; especially for with *P. pastoris* strains. Clone stability can be confirmed by testing productivity over a large number of generations under steady states (about 20–30 residence times) in chemostat cultures growing under a steady state. Alternatively, it can also be assured with periodic changes in dilution rate or the aeration conditions (Cankorur-Cetinkaya et al., 2018). Additionally, and concretely for multi-copy strains, the gene dosage conservation in continuous cultures maintained over long periods is important to be checked.

### Challenges to Be Addressed in *Pichia Pastoris* Continuous Cultivations

An important requirement with continuous bioprocesses is to develop effective alternative promoters to replace the classical P_AOX1_, which is widely used efficiently in fed-batch processes but is not recommended for continuous RPP. In fact, identifying strong and constitutively expressed promoters, and testing them in continuous cultures currently still remains a major goal (Cankorur-Cetinkaya et al., 2018). Obviously, efficiently exploiting the potential advantages of continuous cultivation requires integrating upstream and downstream bioprocess flows. Thus, the implementation of new approaches is a challenge for biotechnology companies in order to reach short process development times jointly with minimal risks in the context of possible hard quality/regulatory requirements (Rathore et al., 2015). At this point a question arises: is continuous cultivation a real alternative to fed-batch cultures for its use in industrial biotechnology based on the *Pichia pastoris* cell factory?

Gasser and Mattanovich (2018) reported some interesting guidelines to transform this yeast from an efficient cell factory to a useful chassis for the production of recombinant proteins and biochemicals as a real alternative to *Saccharomyces cerevisiae*. Furthermore, Moser et al. (2017) investigated the impact of long-term carbon source adaptation toward improved cell growth in RPP processes with *P. pastoris* in the context of Adaptive Laboratory Evolution (ALE). Adaptation of the yeast to growth on methanol over 250 generations was examined and observed a complex correlation among carbon source, cell growth and RPP. Increased specific growth rates on rich and minimal growth media was studied at the level of both population and single clone. Selected clones displayed strain-dependent variations for the yield of P_*AOX1*_-based recombinant protein expression, one showing up to 2-fold increase in terms product titer.

From a bioprocess engineering perspective, process variability often results in issues arising from changes in critical process parameters (CPPs), key parameters of interest (KPIs), or critical material attributes (CMAs), all of which in turn affect critical quality attributes (CQAs). CPPs can change throughout a culture and require using appropriate control strategies to reduce process variability. Advances in process analytical technology (PAT) and quality by design (QbD) approaches have substantially reduced times for process development. According to Hernandez (2015), in the context of transition to continuous bioprocesses it is essential to adapt these methodologies from batch to continuous cultures.

It has been stated that continuous bioprocesses require real-time monitoring of process variability in order to identify eventual deviations and to ensure consistent performance (Rajamanickam et al., 2018). Variability in KPIs (e.g., cell growth, yields, production rates) is especially important because it has a direct impact on process performance and product quality. Thus, continuous bioprocesses require real-time monitoring with standard sensors for direct measurements, or soft sensors that allow the indirect determination of parameters ensuring a high bioprocess efficiency. Recent advances in the monitoring of various cell factories, including soft-sensors, have been reviewed by several authors (Valero and López-Santín, 2017; Veloso and Ferreira, 2017; Randek and Mandenius, 2018). Nevertheless, most of the reported monitoring applications for *P. pastoris* have been described for batch and fed-batch operations. Besides the common sensors used for the monitoring and control of culture conditions and gas analysis, just scarce applications have been reported for continuous mode (Jungo et al., 2007a; Fazenda et al., 2013). In this scenario, further implementing PAT and QbD in *P. pastoris* continuous cultures has been deemed essential for industrial processes.

### How Far Is the Implementation of *Pichia pastoris* Continuous Manufacturing Processes?

Several RPP industrial processes are currently performed with *P. pastoris*. Including both industrial enzymes and biopharmaceuticals, the www.pichia.com website provides a list of products manufactured with *Pichia* which are on the market of late stage development. However, according to the open literature, there are no reported in detail continuous biomanufacturing processes implemented with *P. pastoris*. Nevertheless, taking into consideration the state of the art discussed in this section, some long terms continuous examples described in the literature could eventually become interesting candidates for continuous manufacturing with *P. pastoris*.

A chemostat process producing recombinant hepatitis B small surface antigen (rHBsAg) was maintained working for 2 weeks reaching similar levels of product titer than at the end of a fed-batch culture. Specifically, Q_V_ and *q*_*P*_ were, respectively, about 1.5 and 1.3 times higher, than in fed-batch mode. Interestingly, no contamination issues or/and genome instability were detected after those 2 weeks of continuous operation (Rahimi et al., 2019). In another study, the performance of continuous cultures designed as a combination of turbidostat/chemostat modes at constant cell concentration was evaluated (Wang et al., 2012). As a relevant outcome, it was reported that feeding the system with methanol resulted in high recombinant polygalacturonate lyase PGL expression and a substantial performance improvement relative to conventional fed-batch cultures. Moreover, a chemostat producing lipase B from *Candida antarctica* (CalB) was running 6 weeks at μ = 0.14 h^−1^. In this case, considering the unproductive time spent in setting up, draining out, cleaning and reassembling in fed-batch operation, the overall CalB production was 5.8 times greater than the fed-batch process (de Macedo Robert et al., 2019).

**Figure 3:**
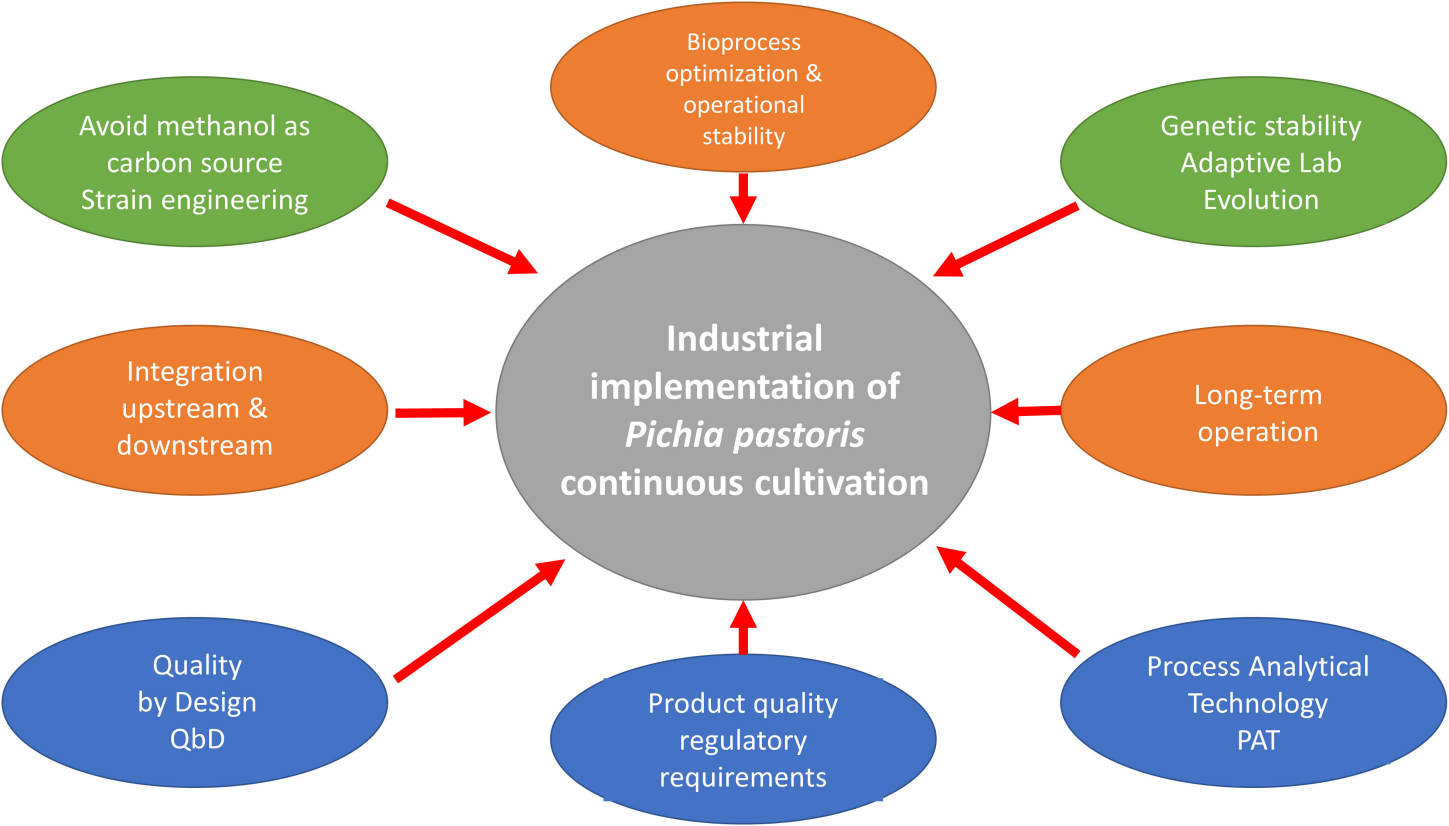
Mean features in the industrial implementation of *Pichia pastoris* continuous cultivation.

Finally, a summary including the main items required to address toward the industrial implementation of continuous cultivation with the cell factory *P. pastoris* is presented in Figure 3.

## Conclusions

The yeast *P. pastoris* is considered an outstanding cell factory alternative for its industrial use in production processes of both metabolites and recombinant proteins. Despite the excellent features of this host, in general, most of the bioprocesses based on fermentation technology are considered still far from achieving the maturity of other industries. In this sense, the present trends toward their optimization are provided by two complementary approaches: Strain development and bioprocess engineering. In order to achieve relevant progresses in the long road to bioprocess optimization it is essential to acquire robust and reliable knowledge of the system used. This knowledge is considered with the objective to be able to design and implement rational developments from any of the both approaches mentioned. In this context, continuous cultivation emerges as an excellent tool to accurately characterize the performance of the cell factory in terms of physiological and production parameters. Furthermore, the steady state conditions achieved during the continuous cultures and the high degree of cell population homogeneity makes this operational mode, an excellent tool toward the development of a wide range of systems biology studies including transcriptomics, proteomics, metabolomics and other cutting edge “-omics” studies.

**Figure 4:**
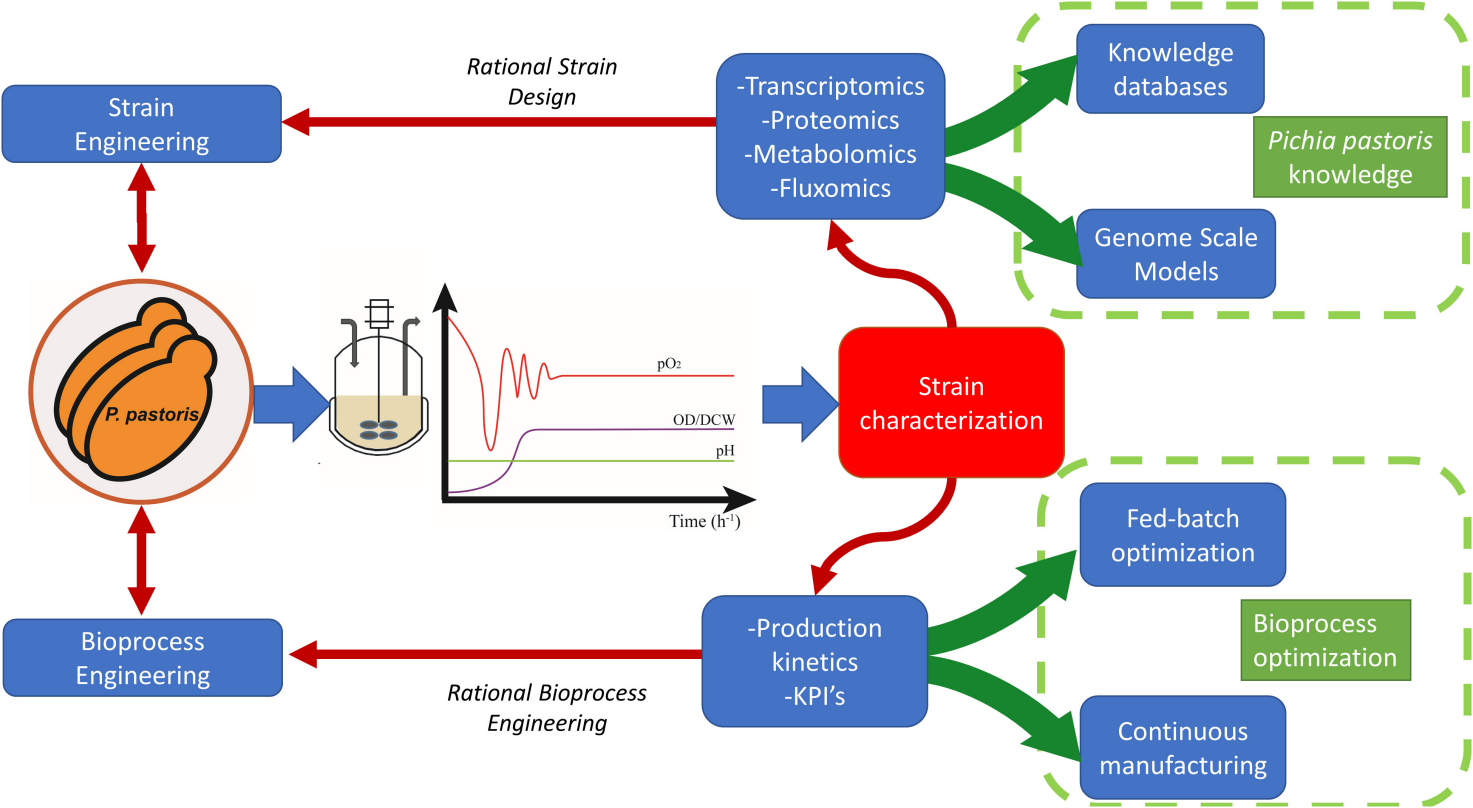
Use of continuous cultivation as a tool toward the rational bioprocess development with *Pichia pastoris* cell factory by combining strain and bioprocess engineering.

In Figure 4 is summarized how continuous cultivation can be used as a tool toward the rational development of bioprocesses with *P. pastoris*. It illustrates the different approaches that, based on the results obtained in continuous cultivation (chemostat), allow to achieve rational improvements at both strain and bioprocess engineering level.

Besides, the current trends of industrial biotechnology is promoting the transition from batch/fed-batch-based processes toward the continuous biomanufacturing due to the numerous advantages that present this operation mode in terms of ensuring a robust and very high product quality as well as, among others, to reduce running costs and minimizing equipment requirements. The potential implementation of biomanufacturing processes with *P. pastoris* has the most relevant challenges with the identification of efficient and methanol-free alternative promoters, the integration of upstream and downstream, reaching short process development times with minimal risks on quality/regulatory requirements through PAT and QbD approaches and, finally, to evaluate the impact of long-term continuous operation on genetic stability and cell physiology.

## Author Contributions

MN-T and XG-O contributed writing the Abstract and the Introduction sections. MN-T and JA wrote the section: Continuous cultivations provide an excellent tool for systems biology research. JM-S and FV wrote the section: Bioprocess characterization with chemostat cultures. XG-O, JM-S, and FV wrote the section: Is continuous cultivation a real alternative for its use in industrial Pichia pastoris processes? XG-O, JM-S, and FV wrote the Conclusions section. All authors read, reviewed, and approved the submitted version.

## Conflict of Interest

The authors declare that the research was conducted in the absence of any commercial or financial relationships that could be construed as a potential conflict of interest.
